# A Manual of Midwifery

**Published:** 1906-09

**Authors:** 


**A Manual of Midwifery.** By Henry Jellett, M.D. Pp. xxv., 1158. London: Baillière, Tindall & Cox. 1905.

This we consider to be one of the best manuals of midwifery we have had to deal with. It gives a very concise and accurate account of the anatomy of the female pelvis, and of the various stages of the development of the ovum; the action of the "trophoblast" and its part in the production of the spaces occupied by the maternal blood sinuses is described clearly and without complication. The part which this structure plays in the changes which occur in the decidua, as shown by the most recent researches, is clearly recognised. The chapter on obstetrical asepsis is excellent; we would recommend its perusal to some of those who have been taking part in the correspondence on modern midwifery in the recent numbers of the *British Medical Journal.*

We note, however, two to us rather important omissions. The author might well have emphasised the importance of making mercurial solutions with *hot* boiled water. This lowers the surface tension of the solution, and renders the antiseptic not only more powerful in its action, but prevents the weakening of the solution which takes place when water is used containing lime. The other point is the enormous increase and the germicidal power of the solution obtained by adding spirit to it. We are aware that this is not always possible, but it should be done in all cases in which spirit can be obtained in sufficient quantities. The author, rightly in our opinion, deprecates the use of the douche as a routine measure, reserving its use for exceptional cases.

The chapter on heart disease as a complication of pregnancy is a most instructive one. The author does not hold that in cases of mitral stenosis chloroform anæsthesia is contra-indicated, and in our opinion makes a very strong case for his view as to its advantages. The value of venesection in these cases, if untoward symptoms arise, he rightly emphasises. In dealing with eclampsia he quotes a table from Lohlein, which seems to show that post-parturient eclampsia is a good deal more dangerous to multiparæ than to primiparæ; this accords with our experience, although we believe the contrary opinion is widely held. His views on the subject represent those of the most recent workers in this field. The views expressed as to the treatment of accidental hemorrhage are those advocated in the Rotunda Hospital, and are shown to be based upon wide experience and sound reasoning.

The author's remarks on "rigid" os as the bugbear of the too conscientious obstetrician who found that it occurred with great frequency when very numerous vaginal examinations were made are quite true. He, however, does not notice that the condition is also associated in some cases with adhesion of the membranes to the lower uterine segment. The chapter on the treatment of contracted pelvis is particularly well written, and lays down valuable rules as to the indications for different methods of treatment. The chapters on the feeding of the infant are also excellent; we should, however, have wished that the author had alluded to the variations in the quality of the maternal milk produced by variations in the intervals between the times of feeding.

We can most strongly recommend the book as a thoroughly interesting and up-to-date account of the subject. It is well written, shows above all things strong common sense, and is clear and concise. The advantage of having the statistics and material of an immense Maternity like the Rotunda to draw upon is enormous, and the book reflects the best opinions and teaching of that justly famed institution.

